# Best Case/Worst Case: ICU (COVID-19)—A Tool to Communicate with Families of Critically Ill Patients with COVID-19

**DOI:** 10.1089/pmr.2020.0038

**Published:** 2020-04-30

**Authors:** Margaret L. Schwarze, Amy Zelenski, Nathan D. Baggett, Elle Kalbfell, Fiona Ljumani, Ethan Silverman, Toby C. Campbell

**Affiliations:** ^1^ Department of Surgery, University of Wisconsin-Madison, Madison, Wisconsin, USA.; ^2^ Department of Medicine, University of Wisconsin-Madison, Madison, Wisconsin, USA.

During the COVID-19 pandemic, critically ill patients have been hospitalized and strictly isolated. In March 2020, palliative care clinicians at the University of Wisconsin were asked to bridge a gap in communication between patients' families and critical care teams, as bedside demands overwhelmed the critical care team's capacity to provide consistent communication with family. In response, we adapted an established intervention, called Best Case/Worst Case, to support daily conversations between hospital-based clinicians and out-of-hospital family.

Our original Best Case/Worst Case (https://tinyurl.com/hfej6hv) intervention was designed specifically for face-to-face clinical interactions to support shared decision making in the context of life-limiting surgical illness. The essential elements of this tool are narrative descriptions, called scenario planning,^1^ and a hand-written graphic aid to illustrate a choice between treatments and to engage patients in deliberation. We have previously tested Best Case/Worst Case with surgeons and acutely ill surgical patients and found that this intervention positively transforms the structure of the decision-making conversation and improves shared decision making on objective measures.^2^

To expand the use of scenario planning to the intensive care unit (ICU) where the patient's trajectory evolves over time, we developed a novel version of Best Case/Worst Case for use with older adults with traumatic injury.^3^ The graphic aid has two sides: Side 1 shows major events and how they are related to the “Best Case Scenario,” specifically, what we are hoping for if everything goes well. Daily updates demonstrate how major events change the patient's overall trajectory and the range of uncertainty between the “Best Case Scenario” and the “Worst Case Scenario,” that is, what we are worried about^4^ (Fig. 1). Side 2 provides an opportunity for families to share what is important to their loved one. This novel version has been tested in Texas, Oregon, and Wisconsin (analysis in progress).

**FIG. 1.**
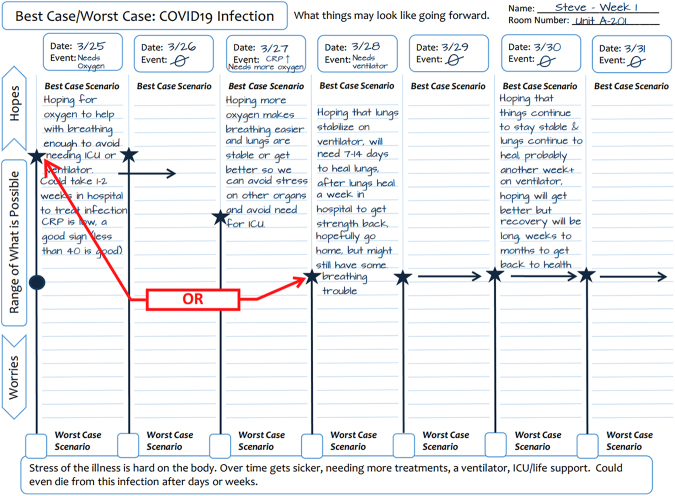
Use of the Best Case/Worst Case: ICU (COVID-19) tool to demonstrate the clinical progression of illness over time for patients receiving critical care. As time goes on, the placement of the star helps you visually show how major events affect the “Best Case” story, for better or worse. The red instructions box shows that the star can be placed anywhere on the line; higher when there is more optimism *or* lower as the patient's condition declines. The oval represents the most likely scenario that is not discussed each day but at times when more targeted prognostication is requested or needed for decision making. ICU, intensive care unit.

Our implementation strategy for Best Case/Worst Case: ICU (COVID-19) includes daily completion of the ICU-tested graphic aid in collaboration with the critical care team followed by telephone calls to family. An essential component of this communication is photo documentation and electronic delivery of the graphic aid to the family so they can follow along. The graphic aid is posted on the patient's door, allowing other care providers to quickly understand the patient's overall trajectory—important when there is high turnover between teams—and learn more about who the patient was before they became sick.

Over the first four weeks of use, response to this intervention has been universally positive. Palliative care clinicians note ease of use; limited time and resources are required to complete the graphic aid and the tool offers a structure to facilitate difficult conversations under extraordinary circumstances. Critical care team members appreciate the connection forged with family, and family members are particularly enthusiastic to see the graphic aid displayed outside the patient's room so clinicians can learn more about who their loved ones were before their current illness. As supplies of personal protective equipment dwindle, we anticipate this could also bridge the physical distance between the critical care team and “remote PalCare support.”

A toolkit with step-by-step instructions, blank graphic aids, and examples for use can be downloaded, free of charge, at (https://tinyurl.com/bcwc-covid19).

## Abbreviation Used

ICU: intensive care unit
